# Sarcopenia and Hearing Loss in Older Koreans: Findings from the Korea National Health and Nutrition Examination Survey (KNHANES) 2010

**DOI:** 10.1371/journal.pone.0150281

**Published:** 2016-03-15

**Authors:** Jieun Lee, Kyungdo Han, Jae Jun Song, Gi Jung Im, Sung Won Chae

**Affiliations:** 1 School of Medicine, Korea University, Seoul, Korea; 2 Department of Biostatistics, Catholic University of Korea, School of Medicine, Seoul, Korea; 3 Department of Otolaryngology-Head and Neck Surgery, Korea University College of Medicine, Seoul, Korea; West Virginia University School of Medicine, UNITED STATES

## Abstract

Age-related hearing impairment (ARHI) is becoming a more significant issue as geriatric population increases. Sarcopenia in older people is known to have a diverse health problem in various circumstances in recent studies. We assessed whether the decrease in muscle mass is related to ARHI. We used the 2010 data of the Korea National Health and Nutrition Examination Survey (KNHANES) to examine the associations between sarcopenia and ARHI. A total number of participants was 1,622 including 746 males and 876 females aged 60 years or older. Muscle mass was assessed as an appendicular skeletal muscle mass, and hearing loss was defined as the pure-tone averages (PTA) of test frequencies 0.5, 1, 2, 4 kHz at a threshold of 40 dB or higher in worse hearing side of the ear. Among 1,622 participants, 298 men and 256 women had hearing loss. Appendicular muscle mass (ASM), expressed as kg, was categorized in tertiles. In female population, after adjusting for age, smoking, drinking, amount of exercise, total body fat, education level, income level, and tinnitus, the odds ratio (OR) for hearing loss was 1.57 (95% confidence interval (CI) = 0.92–2.68) in the middle tertile and 1.79 (1.03–3.08) in the lowest tertile, compared with the highest tertile. P for trend in this model was 0.036. Controlling further for hypertension, diabetes mellitus, chronic kidney disease, and three types of noise exposure did not change the association. Larger muscle mass is associated with lower prevalence of hearing loss in elderly Korean females.

## Introduction

Age-related hearing impairment (ARHI), also known as presbycusis, is the most common sensory dysfunction in adults [1]. This condition is a sensorineural hearing loss that can have a negative effect on a person’s quality of life by impairing effective communication and thus, cause social isolation [2, 3]. ARHI is going to be a more important public health issue in the future as the elderly population is expected to increase by more than 2-fold in the next 20 years in Korea [4].

There have been some studies on the risk factors of hearing impairment such as Body Mass Index (BMI), waist circumference, adipose tissue volume and environmental risk factors as smoking, noise and alcohol consumption. Having a high BMI has been regarded as a risk factor for hearing impairment [5, 6], and larger waist circumference was also found to be an independent risk factor of ARHI [7]. High visceral adipose tissue was found to be associated with the high prevalence of hearing impairment among elderly Korean women [8]. Occupational noise and smoking were found to be risk factors for ARHI, whereas moderate alcohol consumption seemed to have a protective effect [5]. If we know more about the potentially modifiable risk factors, we could prevent or delay the onset of ARHI.

In recent studies, sarcopenia in older people turned out to have an adverse health effect in various circumstances. Sarcopenia is an age-associated loss of muscle mass. Among the people aged 60–70 years, 5–13% of them have low muscle mass, and this prevalence increases to almost 50% in people over 80 years old [9, 10]. The European Working Group on Sarcopenia in Older People (EWGSOP) defined sarcopenia as a syndrome which brings out adverse outcomes including physical disability, death and poor quality of life [11]. Sarcopenia was associated with cardiovascular risk factors such as arterial stiffness [12], and it is also known to worsen metabolic impairments [13].

Both BMI and waist circumference are well known predictors of developing metabolic impairments. Obesity is also known as a risk factor for ARHI, whereas the audiometric association with sarcopenia in elderly population remains unexamined. Given the fact that both sarcopenia and obesity are associated with metabolic impairments, we assumed that the association between sarcopenia and ARHI has clinical significance.

We supposed that Sarcopenia is related to the larger prevalence of age-related hearing impairment, and we used the Korea National Health and Nutrition Examination Survey (KNHANES) Ⅴ (2010–2012) data to support the hypothesis.

## Materials and Methods

### Study Population

The Korea National Health and Nutrition Examination Survey (KNHANES) Ⅴ (2010–2012) is the fifth cross-sectional survey for the South Korean population conducted by the Korean Ministry of Health and Welfare since 1998. This survey comprises a health interview survey, a health examination survey, and a nutrition survey. Each year’s survey of KNHANES is independent and represents the South Korean population based on its rolling sample design. We used 2010 data of KNHANES which is comprised of 8,958 South Koreans. We excluded people who were younger than 60 years old (n = 6,930). Among people aged 60 years or older, we excluded people who only tested for either hearing loss or muscle mass (n = 381). We also excluded 13 people who had liver diseases such as hepatitis B, hepatitis C, and liver cirrhosis, and 12 people who had chronic kidney diseases with GFR<30, because these diseases can affect people’s body composition. A total of 1,622 subjects (746 males and 876 females) were included in the analysis.

### Assessment of Muscle Mass

Muscle mass in each subjects was assessed as appendicular muscle mass (ASM) measured by dual-energy x-ray absorptiometry (DXA) (QDR 4500A, Hologic Inc., Waltham, MA, USA). ASM (kg) was derived as the sum of the lean soft tissue mass of the upper and lower appendixes (arms and legs), after the method of Heymsfield et al. [14].

Muscle mass was categorized in tertiles: t1 (subjects whose ASM<18.6 in men and <12.64 in women), t2 (subjects whose ASM is 18.6–20.85 in men and 12.64–14.24 in women), and t3 (subjects whose ASM≥20.85 in men and ≥14.24 in women).

### Assessment of Hearing Loss

Pure-tone audiometry, the gold standard for hearing loss evaluation, was conducted by a SA 203 audiometer (Entomed; Malmö, Sweden) in the study population. The test was conducted in a soundproof booth and the instruction was given by certain otolaryngologists trained to operate the audiometry. Subjects used supra-auricular headphones in a soundproof booth, and only air conduction was measured. Study subjects pushed a button when they heard a tone. Test frequencies were 0.5, 1, 2, 3, 4, and 6 kHz.

**Fig 1:**
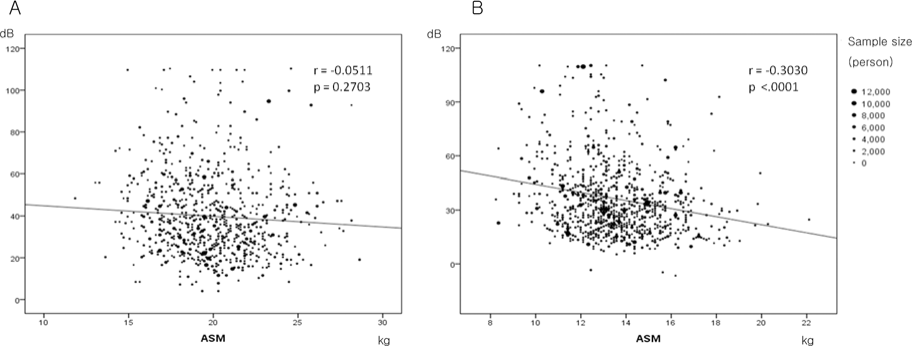
**Distribution of the maximum hearing threshold (dB) according to ASM in male (A) and female (B) subjects.** Larger ASM was inversely associated with the maximum dB (p = 0.2703 in males and p = < .0001 in females).

Hearing loss was defined as the pure-tone averages (PTA) of test frequencies at 0.5, 1, 2, 4 kHz at a threshold of 40 dB or higher in worse hearing side of the ear. In Fig 1, the maximum dB was defined as the higher value between the mean threshold of hearing level in right and left ear.

### Assessment of Covariates

Information on the study population including BMI, waist circumference, total body fat percentage, smoking, alcohol consumption, exercise level, education level, household income, noise exposure, stress level, and medical conditions were obtained from the result of KNHANES. BMI was calculated based on the data of height which was measured to the nearest 0.1cm with a stadiometer (Seca 225; Seca, Hamburg, Germany) and weight which was measured using an electronic scale (GL-6000-20; CasKorea, Seoul, Korea) with an accuracy of 0.1kg. Waist circumference was measured using a measuring tape (Seca 200; Seca) to the nearest 0.1cm.

Hypertension was defined as a systolic blood pressure (BP)≥140mmHg, a diastolic BP≥90mmHg, or treatment with antihypertensive agents. Blood pressure was measured three times with a mercury sphygmomanometer (Baumanometer; Baum, Copiague, NY, USA), and the average of the second and the last measurements became each subject’s final blood pressure. Diabetes mellitus (type 2) was defined by a fasting plasma glucose level≥126mg/dL, treatment with oral hypoglycemic agents or insulin, or diagnosis by a physician. Chronic kidney disease was defined as estimated glomerular filtration rate (GFR)<60mL/min/1.73m^2^, and the GFR was calculated based on the formula of the Modification of Diet in Renal Disease Study.

For certain items such as smoking, alcohol consumption, exercise level, education level, household income, noise exposure, stress level, and medical conditions such as melancholy, tinnitus, and dizziness, all participants were asked to fill out the self-reported questionnaires. In terms of smoking, the participants were categorized into current smokers and the others. A participant was considered as a ‘drinker’ if he/she drank more than once per month over the past year. A strenuous physical activity done for at least 20 minutes at a time and more than three times a week was considered as a regular exercise. Education level was categorized into two groups: longer than 9 years of education (finished beyond middle school) and the others. The lowest quartile of household income was considered as low income. ‘Noise at work’ was defined as the work experience of more than 3 months at the place where people have to speak up to communicate with one another. ‘Noise outside work’ was defined as the experience of more than 5 hours per week outside workplace where people have to speak up to communicate with one another. ‘Noise at any given moment’ was defined as the exposure to a very loud noise such as gunfire noise. The subjects answered ‘yes’ if they think they experience a lot of stress in everyday life. Depressed mood for more than 2 weeks that affects everyday life in the past year was considered as melancholy. In terms of tinnitus and dizziness, the subjects answered ‘yes’ to each question if they have experienced each condition in the past year.

### Statistical Analysis

The data in Table 1 are presented as mean±SE for continuous variables or as % (SE) for categorical variables, and t-tests or χ^2^ tests were done, respectively. The association between ASM and PTAs of 0.5, 1, 2, 4 kHz was presented as Pearson’s correlation, and age-adjusted linear regression was used to verify the relationship between body composition variables and PTAs. Multiple logistic regression analyses were also performed to estimate the association between ASM and hearing loss. And we presented as OR (95% CI) after adjusting for certain factors in each model using hierarchical analysis (model 1: adjusted for age; model 2: adjusted for age, current smoking, alcohol use, regular exercise, and body fat percentage; model 3: adjusted for age, current smoking, alcohol use, regular exercise, body fat percentage, education level, income level, and tinnitus; model 4: adjusted for all variables in model 3 plus hypertension, diabetes mellitus, and chronic kidney disease; model 5: adjusted for all variables in model 4 plus noise at work, noise outside work, and noise at any given moment). All statistical analyses were stratified by gender and performed using the SAS survey procedure (version 9.3; SAS Institute, Cary, NC, USA) to reflect the sampling weights and complex sampling design analyses of KNHANES. All data of P < 0.05 were accepted as statistically significant results.

**Table 1:** Clinical characteristics of study population. Data are presented as mean±SE or as % (SE). BMI, body mass index; ASM, appendicular skeletal muscle mass; Ht, height; Wt, body weight.

	Male			Female		
	HI(-)	HI(+)	p-value	HI(-)	HI(+)	p-value
	(n = 448)	(n = 298)		(n = 620)	(n = 256)	
Age (yr)	66.8±0.3	70.6±0.4	< .0001	68.4±0.3	73.0±0.6	< .0001
BMI (kg/m^2^)	23.6±0.2	23.3±0.2	0.0198	24.5±0.2	23.8±0.2	0.2548
Waist circumference (cm)	85.8±0.5	85.2±0.6	0.2555	83.6±0.4	82.7±0.7	0.4108
ASM (kg)	20.1±0.2	19.8±0.2	< .0001	13.8±0.1	12.9±0.1	0.1475
ASM/Ht^2^ (kg/m^2^)	7.3±0.0	7.2±0.1	0.0025	5.9±0.0	5.7±0.1	0.1685
ASM/Wt (%)	31.0±0.2	31.0±0.2	0.4870	24.4±0.2	24.2±0.2	0.9623
Total body fat (%)	23.6±0.4	23.6±0.4	0.9473	35.4±0.4	35.2±0.4	0.6278
Current smoker (%)	25.4 (2.3)	24.3 (3.0)	0.8023	2.3 (0.6)	7.9 (2.4)	0.0009
Drinker (%)	68.3 (2.5)	54.2 (2.9)	< .0001	21.2 (2.2)	20.1 (2.8)	0.7270
Regular exercise (%)	25.6 (2.8)	22.4 (3.2)	0.3805	17.2 (1.8)	17.5 (2.9)	0.9024
Education (>9 y) (%)	43.3 (3.4)	28.5 (3.1)	0.0014	11.4 (1.5)	3.8 (1.2)	0.0002
Low income (lowest quartile) (%)	28.0 (2.6)	47.1 (3.4)	< .0001	50.0 (2.7)	47.9 (4.1)	0.6692
Regions, urban area (%)	70.3 (5.1)	65.0 (5.8)	0.2902	64.9 (4.9)	61.5 (5.8)	0.4738
Stress (yes) (%)	14.5 (2.2)	10.6 (2.2)	0.2147	28.4 (2.2)	29.8 (3.3)	0.7162
Melancholy (yes) (%)	8.8 (1.6)	11.5 (2.3)	0.3322	18.5 (1.9)	20.0 (2.8)	0.6915
Tinnitus (yes) (%)	22.9 (2.3)	39.4 (4.0)	0.0006	25.4 (2.1)	43.9 (3.3)	< .0001
Dizziness (yes) (%)	10.0 (1.7)	20.5 (3.7)	0.0023	20.9 (2.6)	37.0 (4.0)	< .0001
Hypertension (%)	56.1 (2.7)	56.5 (3.8)	0.9269	65.5 (2.2)	67.1 (4.0)	0.7354
Diabetes mellitus (%)	19.4 (2.7)	18.2 (2.4)	0.7430	19.2 (1.9)	25.3 (3.8)	0.1167
Chronic kidney disease (%)	8.2 (1.6)	19.6 (3.0)	0.0004	14.9 (1.8)	23.8 (3.4)	0.0125
Noise at work (yes) (%)	14.9 (2.0)	20.2 (2.8)	0.0806	6.0 (1.2)	10.4 (2.5)	0.0500
Noise outside work (yes) (%)	1.5 (0.5)	3.1 (1.4)	0.0971	1.7 (0.7)	1.3 (0.7)	0.7348
Noise at any given moment (yes) (%)	45.7 (4.2)	41.5 (4.6)	0.3825	8.0 (1.6)	11.6 (3.0)	0.1202

## Results

Characteristics of the subjects are shown in Table 1. In both males and females, the mean age was significantly higher in those with hearing impairment than those without it (all p < .0001). Males with hearing impairment had significantly smaller ASM than those without it (p < .0001). In case of females, those with hearing impairment also had smaller ASM than those without it but the association was insignificant (p = 0.1475). Percentage of current smokers was significantly higher in females with hearing impairment than that in females without it (p = 0.0009), but this association was not observed in males. Percentage of people who have educational background beyond middle school was significantly lower in both males and females with hearing impairment (p = 0.0014 and 0.0002, respectively). Percentage of people whose income level is in the lowest quartile was significantly higher in males with hearing impairment than those without it (p < .0001). In case of females, percentage of people whose income level is in the lowest quartile was lower in those with hearing impairment than those without it, but the difference was insignificant (p = 0.6692).

Association with ASM and the maximum dB in males and females is shown in Fig 1. Larger ASM was inversely associated with the maximum dB in males (r = -0.0511), but the trend was insignificant (p = 0.2703). In case of females, those with larger ASM showed smaller value in maximum dB, which means better hearing (r = -0.3030) and it was significant (p < .0001).

Age-adjusted linear regression analysis between hearing loss and anthropometric parameters is shown in Table 2. In female population, hearing loss was correlated negatively with ASM and ASM/Ht^2^ (p = 0.0017 and p = 0.0338, respectively). The correlation between hearing impairment and other factors were statistically insignificant in males.

**Table 2:** Age-adjusted linear regression between hearing loss and anthropometric parameters. BMI, body mass index; WC, waist circumference; ASM, appendicular skeletal muscle mass; Ht, height; Wt, body weight; FAT, total body fat percentage.

	Beta (SE)	p-value
	Male	
BMI (kg/m^2^)	0.296046 (0.316)	0.3508
WC (cm)	0.014816 (0.088)	0.8667
ASM/Ht^2^ (kg/m^2^)	1.067584 (1.097)	0.3321
ASM/Wt (%)	- 0.053898 (0.275)	0.8447
ASM (kg)	0.302832 (0.359)	0.4001
FAT (%)	0.096336 (0.160)	0.5481
	Female	
BMI (kg/m^2^)	- 0.243144 (0.250)	0.3325
WC (cm)	- 0.044782 (0.095)	0.6374
ASM/Ht^2^ (kg/m^2^)	- 2.550579 (1.191)	0.0338
ASM/Wt (%)	- 0.353841 (0.332)	0.2883
ASM (kg)	- 1.167510 (0.365)	0.0017
FAT (%)	0.116143 (0.161)	0.4721

When ASM was categorized in tertiles, smaller amount of muscle mass was significantly associated with hearing impairment in females after adjusting for age (p for trend = 0.0084; Table 3). Controlling further for smoking, drinking, exercise, and the amount of total body fat (model 2) did not change the association (p for trend = 0.0229). Further adjustment for education level, income level, and tinnitus (model 3) attenuated the association but it remained statistically meaningful. The OR for hearing impairment was 1.57 (95% CI = 0.92–2.68) in the middle tertile and 1.79 (95% CI = 1.03–3.08) in the lowest tertile, compared with the highest tertile. (p for trend = 0.036). This association remained significant even after further adjusting for hypertension, diabetes mellitus, and chronic kidney disease (p for trend = 0.0407; model 4). Controlling further for three types of noise exposure (noise at work, noise outside work, and noise at any given moment; model 5) did not change the association. In case of males, the association between the amount of muscle mass and hearing impairment was statistically insignificant in all 5 models.

**Table 3:** Adjusted odd ratios (OR) and 95% confidence intervals (CIs) for hearing impairment according to the appendicular muscle mass tertiles. Model 1 is adjusted for age. Model 2 is adjusted for age, current smoking, alcohol use, regular exercise, and body fat percentage. Model 3 is adjusted for age, current smoking, alcohol use, regular exercise, body fat percentage, education level, income level, and tinnitus. Model 4 is adjusted for all variables in model 3 plus hypertension, diabetes mellitus, and chronic kidney disease. Model 5 is adjusted for all variables in model 4 plus noise at work, noise outside work, and noise at any given moment. Smaller amount of muscle mass was associated with hearing impairment in females in all 5 Models. T3; ASM≥20.85 in men and ASM≥14.24 in women, T2; 18.6≤ASM<20.85 in men and 12.64≤ASM<14.24 in women, T1; ASM<18.6 in men and ASM<12.64 in women.

	Model1	Model2	Model3	Model4	Model5
	Male				
T3	1 (ref.)	1 (ref.)	1 (ref.)	1 (ref.)	1 (ref.)
T2	0.68 (0.44–1.05)	0.67 (0.46–1.07)	0.74 (0.48–1.14)	0.83 (0.52–1.31)	0.81 (0.52–1.28)
T1	0.85 (0.54–1.34)	0.89 (0.56–1.41)	0.82 (0.51–1.33)	0.92 (0.55–1.55)	0.97 (0.57–1.62)
p for trend	0.4618	0.6077	0.4203	0.7482	0.869
	Female				
T3	1 (ref.)	1 (ref.)	1 (ref.)	1 (ref.)	1 (ref.)
T2	1.71 (1.05–2.79)	1.61 (0.98–2.64)	1.57 (0.92–2.68)	1.72 (0.94–3.16)	1.69 (0.91–3.14)
T1	1.94 (1.18–3.18)	1.80 (1.08–3.00)	1.79 (1.03–3.08)	1.88 (1.02–3.48)	1.87 (1.01–3.45)
p for trend	0.0084	0.0229	0.036	0.0407	0.0413

## Discussion

In this cross-sectional study, larger muscle mass was associated independently with lower prevalence of hearing impairment in elderly Korean females, and this association was statistically significant even after adjusting for age, smoking, drinking, exercise level, total body fat, education level, income level, tinnitus, hypertension, diabetes mellitus, chronic kidney disease, and noise exposure. This is the first time to reveal the relationship between sarcopenia and ARHI. These findings may suggest possible lifestyle changes to prevent ARHI and even help people delay the onset of ARHI.

Low level of physical activity is one of the causes of sarcopenia, along with inflammation, changes in endocrine function, chronic disease, and nutritional deficiencies [15]. Even though sarcopenia is a multifactorial syndrome, physical activity seems to play a key role [11].

In recent studies, physical exercise appears to induce neuroprotection by improving synapse density and neuronal complexity, and it also appears to restore hippocampal neurogenesis after irradiation in rodents [16, 17]. Brain-derived neurotrophic factor (BDNF) is a neurotrophin which plays an important role in maintaining the healthy neurons [18]. BDNF promotes the survival of spiral ganglion neurons (SGNs), as proved in vitro [19, 20] and in vivo [21, 22]. Exercise increases the serum BDNF level [17], and this also appears to promote elevated brain BDNF levels in rats [23]. Physically inactive people are prone to be in sarcopenic condition and to produce less BDNF, which may subsequently increase susceptibility to the age-related neurodegeneration that will lead them to have ARHI.

Oxidative stress may also be a potential explanation for the association of sarcopenia with ARHI. Cochlea is sensitive to the changes in arterial blood supply as the stria vascularis is highly vascularized [8]. Interruption of blood flow to the cochlea detrimentally affected cochlear function in gerbils [24], and rabbits [25, 26], and this association also suggested in older adult humans [27]. People who are engaged in higher levels of physical activity may have better vascular supply to the cochlea [28], and less probability of having auditory neurodegeneration and neurotransmitter loss [29]. Physical activity also causes endothelium-mediated arterial vasodilation, and enhances blood flow to the stria vascularis [30]. Sarcopenia itself was also found to be significantly associated with poor blood flow by greater arterial stiffness, particularly in females [31]. Greater muscle mass requires more blood supply, and it results in a higher cardiac output, stroke volume and size adaptation of the arteries [32]. Thus, sarcopenic patients may be more prone to have greater arterial stiffness in smaller diameter arteries. This may also lead to the reduction in cochlear blood flow, and as a result, decreased blood supply to the cochlea can trigger poorer hearing threshold.

In our study, larger muscle mass was associated independently with lower prevalence of hearing loss in elderly Korean females. This association was not shown in Korean males. In general, men tend to have greater muscle mass and physical performance compared with women [33, 34]. When we assume that sarcopenia is a risk factor of ARHI, it may be possible to speculate that there is a potential cutoff point in muscle mass which from a certain point, having smaller muscle mass does not associated with ARHI because in that group, people already have sufficient ‘preventive’ amount of muscle mass for ARHI.

Strengths of our study include its nationally representative data and validated methods to quantify sarcopenia and hearing loss. Our study subjects were comprised of the general population of South Korea and they represented the whole South Korean population as KNHANES is a rolling sample design. Also, KNHANES used the pure-tone audiometric test to whole study population which is a gold standard of hearing tests, and degree of sarcopenia was represented as ASM which was measured by DXA.

There are several limitations in our study. It is difficult to evaluate causal relations between muscle mass and age-related hearing impairment because our study is a cross-sectional study. Therefore, our study invites future prospective studies on sarcopenia and hearing loss, which will allow us to have insight on causal relationship of them. Second, our study used KNHANES data which used self-reported questionnaires to examine respondents’ lifestyle, which can have the recall bias.

Sarcopenia is underdiagnosed and undertreated even though it is very common among elderly people [11]. Paying attention to the sarcopenic condition and putting efforts to prevent this condition may help one to have better hearing later in life, and further studies on sarcopenia and hearing loss may give us insight on the mechanism and the way to prevent ARHI.
